# Magnitude of undernutrition and associated factors among adult tuberculosis patients attending public health facilities in Haramaya District, Eastern Ethiopia

**DOI:** 10.1186/s12890-023-02318-6

**Published:** 2023-01-30

**Authors:** Fasika Tadesse, Habtamu Mitiku, Sagni Girma, Abera Kenay

**Affiliations:** 1 Oromia Regional State Eastern Hararghe Zone Health Office, Harar, Ethiopia; 2 School of Medical Laboratory Sciences, College of Health and Medical Sciences, Haramaya University, Harar, Ethiopia; 3 School of Nursing and Midwifery, College of Health and Medical Sciences, Haramaya University, Harar, Ethiopia; 4 Department of Obstetrics and Gynaecology, Leiden University Medical Centre, Leiden, the Netherlands; 5 Department of Obstetrics and Gynecology, University Medical Centre Groningen, University of Groningen, Groningen, the Netherlands

**Keywords:** Undernutrition, Adult TB patients, Haramaya, Ethiopia

## Abstract

**Background:**

Tuberculosis is one of the top ten causes of illness, death, and disability throughout the world. Undernutrition reduces immunity, which makes latent tuberculosis more likely to become active tuberculosis. Tuberculosis makes these conditions worse. The body of a person suffering from TB has an increased demand for energy, which often causes a TB patient to lose a significant amount of weight and this can worsen acute undernutrition. The aim of this study was to assess the magnitude of undernutrition and its associated factors among adult TB patients in public health facilities in Haramaya district, eastern Ethiopia.

**Methods:**

Institution-based cross-sectional study was conducted among 330 adult tuberculosis patients on follow-up in public health facility of Haramaya District, eastern Ethiopia from January 10, 2021 to February 20, 2021. An anthropometric assessment was done after a face-to-face interview using a pretested structured questionnaire. SPSS 24 was used to analyze the data. Bivariable and multivariable logistic regression model was used to identify factors associated with undernutrition.

**Results:**

The overall prevalence of undernutrition was 43.6% (95% CI 38.2–49.1%). Proportion of severe, moderate and mild undernutrition was 11.8%, 12.4%, and 19.4%, respectively. Age group of 18–24 years (AOR = 4.12; 95% CI 1.36–12.51), not have formal education (AOR = 1.76; 95% CI 1.01–3.08), having large family size (AOR = 2.62; 95% CI 1.43–4.82), low dietary diversity (AOR = 2.96; 95% CI 1.75–4.99), lack of latrine (AOR = 2.14; 95% CI 1.26–3.65), history of TB treatment (AOR = 2.56; 95% CI 1.19–5.54) and taking intensive phase of anti-TB drugs (AOR = 3.18; 95% CI 1.62–6.25) were factors found significantly associated with under nutrition.

**Conclusion:**

The prevalence of undernutrition was high. Age, educational status, family size, dietary diversity, toilet facility, history of tuberculosis medication and intensive phase of anti-TB drugs were found significantly associated with undernutrition. The nutritional derangement could call for fast nutritional intervention in the management of pulmonary tuberculosis patients.

## Introduction

Tuberculosis (TB) is a leading cause of morbidity and mortality worldwide, an estimated 10.6 million people fell ill with TB in 2021, an increase of 4.5% from 10.1 million in 2020. Globally, the estimated number of deaths from TB in 2021 was 1.6 million. TB morbidity and mortality are highest in developing countries. The number of new TB cases recorded in 2021 was 6.4 million [1].

Under nutrition speeds up the progression of TB from infection to active tuberculosis. In addition, under nutrition is associated with increased risk of death and relapse of the disease[2]. Under nutrition also affects the treatment process of tuberculosis [3]. Poor dietary and feeding practices impede the fight against TB especially in developing countries. To make matters worse, TB is accompanied by other diseases that adversely affect the nutritional status. Further, TB medications have side effects that make it hard for the patient to eat [4].

The body of a person suffering from TB has an increased demand for energy, which often causes a TB patient to lose a significant amount of weight and this can worsen acute undernutrition. One study shows that under nutrition among pulmonary TB patients is a result of preexisting chronic undernutrition and concurrent active infection, which increases the severity of weight loss [5].

Patients who are undernourished have diseases that are more severe, which raises the chance of death and severe undernutrition at diagnosis [6]. Evidence suggests that undernutrition in people with active TB is linked to a two- to four-fold increase in mortality and a five-fold risk of drug-induced hepatotoxicity [7].

Tuberculosis is traditionally related to malnutrition, reduced appetite, low dietary intake, malabsorption and redoubled caloric demand [8]. However, this relationship is thought to be bidirectional, because the clinical course of the disease results in secondary malnutrition, and malnutrition is additionally a risk issue for the disease [9].

Malnutrition has a very heavy impact on how the disease progresses [10]. People who take medications like patients on TB medication may experience unpleasant side-effects that can ultimately reduce their dietary intake [11]. Under nutrition may be due to illness that impairs nutrient intake and metabolism, or result from inadequate intake of macronutrients, micronutrients or both [12].

Studies specifically focused on undernutrition in TB patients are scarce in Ethiopia, particularly when one descends to the zonal and district levels. There have been very few reports on prevalence, severity and implications of under-nutrition among TB patients [13, 14]. These studies, however, did not adequately address issues like behavioral factors, food access, the duration of the disease before diagnosis, the history of prior therapy, and hygiene factors. A persistent nutritional problem affects the community in East Hararghe zone and Harames district in particular. This issue has a significant impact on patients with tuberculosis throughout treatment follow-up. In addition, based on earlier evidence obtained from some studies in Ethiopia, there were no interventions such as nutrition counseling and support provided to patients who receiving treatment for tuberculosis. Therefore, the purpose of this study was to assess undernutrition and its associated factors among adult TB patients receiving treatment in public health institutions in the Haramaya district of eastern Ethiopia.

## Methods

### Study design and setting

The study was conducted in Haramaya District, Eastern, Ethiopia from January 10, 2021 to February 20, 2021. Haramaya district is one of the 20 districts of East Hararghe zone. It is 504 km from Addis Ababa, the nation’s capital, and 19 km from Harar. According to population projection, the total population of the district in 2019 was 310,039. The district has 33 rural and one urban kebeles. Nine health centers and one hospital in the district provided TB care, including directly observed therapy short course (DOTS), during the study period. A total of 357 TB patients were receiving follow-up care in 2020.

The study included adult TB patients over the age of 18 who were receiving anti-tuberculosis medication. While those patients who were pregnant, seriously ill, or unable to communicate were excluded.

### Sample size and sampling technique

The sample size was calculated using single population proportion formula (n=(z α/2)^2^ * p(1-p)/ d²). The prevalence of malnutrition among TB patients (27.15%) was obtained from a study conducted in Addis Ababa [15]; the maximum allowable error (margin of error) was 0.05 with a 95% confidence level. With a 10% non-response rate included, the final sample size was 332. Sample size was reached using simple random sampling from 1 hospital and 8 health center.

## Data collection methods

Data on sociodemographic characteristics such as age, sex, religion, ethnicity, marital status, educational status, main occupation, family size, residence and average monthly income; dietary factors such as main source of food for family, nutritional care and support, dietary counseling, individual dietary diversity and household food security; clinical factors such as family history of TB, co-morbidity, HIV co-infection, type of TB treatment, duration of anti-TB medication; and problem with eating (having mouth ulcer, nausea and/or vomiting, poor appetite, or pain or difficulty of swallowing); lifestyle factors such as physical exercise, alcohol consumption, khat chewing and cigarette smoking; environmental factors such as latrine possession, living with domestic animal, drinking water source, living house possession, and time to fetch drinking water were collected using structured questionnaire adapted from reviewing relevant literature (13, 14, 16–18).

Anthropometric measurement (weight, height,) was obtained from all participants by using a standardized procedure. A height measuring board and digital balance, Seka, which is German model for weight measurement was used. The weight was measured on a portable standing scale to the nearest 0.1 kg after regular calibration against known weight. During the procedure, participants were asked to wear light clothing and barefoot. With the use of a measuring tape mounted on a wall, height was measured to the nearest 0.1 cm. Subjects were measured against the wall without shoes and with their heels together, their heads straight ahead and their eyesight on a line parallel to their body (Frankfurt plane).

Body Mass Index (BMI) was computed as body weight in kilograms divided by the square of height in meter. Severe undernutrition is defined as a BMI < 16.0 kg/m^2^, moderate undernutrition as a BMI = 16.0 kg/m^2^-16.99 kg/m^2^, mild undernutrition as a BMI = 17.0 kg/m^2^-18.49 kg/m^2^, Normal weight as a BMI = 18.5 kg/m^2^-24.99 kg/m^2^, Overweight as a BMI = 25.0 kg/m^2^-29.99 kg/m^2^ and Obese as a BMI ≥ 30kg/m2. To make interpretation simpler, we dichotomized the BMI into two categories: undernutrition (BMI < 18.5) and Normal (BMI ≥ 18.5).

Individual dietary diversity of participants was measured by standard individual dietary diversity scale items, using 24 hours’ dietary recall method as per FAO guideline. Low Individual dietary diversity score was considered when adult TB patients consumed less than seven food groups out of 14 food groups and not unless otherwise [19].

To assess household food security, the standard household food security access scale was employed [17]. It consists of nine questions regarding food insecurity, each of the nine items was coded as 0 = no occurrence, 1 = rarely, 2 = sometimes, 3 = often. Hence, the total score could range from 0 to 27. Each of the questions were asked using a four-week (30 days) recall period. Finally, the score was added and dichotomized into two categories: household food insecure and household food secure. A score of two or above shows that there was food insecurity in the household [17].

## Data quality control

The questionnaire was pretested at a comparable health center, Aweday health center, which is not among the sampled health centers, prior to the actual data collection. On 5% of the samples, the questioner was pre-tested, and the response categories were modified as necessary. Data collectors and supervisors underwent a two-day training session on correct questionnaire completion and the use of weight and height scales to reduce inter- and intra-observer errors. The questionnaire was written in English, translated into Afan Oromo, and then back into English by a person with proficiency in the English language. The data collectors were closely supervised to ensure high-quality data was collected. The principal investigator reviewed each questionnaire to make sure the questions had been correctly filled. In a subsample that was chosen at random (5%), the data were reviewed again.

## Data processing and analysis

Data were coded and entered in EpiData 3.1 then exported to SPSS version 23 for analysis. Descriptive statistics were used to describe study participants using figures and tables. Multi-collinearity test was carried out to see the correlation between independent variables by using collinearity statistics. Variance inflation factors > 5 was considered as suggestive of existence of multi-collinearity. In this study multi-collinearity was not detected between the independent variables. Goodness of fit was checked by Hosmer–Lemeshow test. The full model, including all the 12 variables selected to be included in multivariable logistic regression analysis, well fit actual data. Bivariable and multivariable logistic regression analyses were conducted to assess factors associated with undernutrition. Association was described using odds ratio along with their 95% confidence interval. Variables with p < 0.25 in the bivariable analysis were considered as a candidate for multivariable logistic regression. Finally, p < 0.05 was considered as significantly associated factors with TB patients.

## Results

### Sociodemographic characteristics of respondents

From 332 adult TB patients selected for this study, 330 participated with response rate of 99.4%. Majority (79.1%) of the participants were rural residents. The mean age was 29.4 (± 9.5) years, ranging from 18 to 65 years. More than half (58.5%) of the participants were males. More than three-fourth (77.9%) of participants were  married. Two hundred and fourteen (64.8%) participants had no formal education and 257(77.9%) were living in the family with > 5 person in the household with a median family size of 4 (Table 1).

**Table 1:** Socio-demographic characteristics of adult tuberculosis patients on follow up at public health facilities of Haramaya district, eastern Ethiopia, 2021 (n = 330)

Characteristic	N	%
Residence		
Urban	69	20.9
Rural	261	79.1
Age (in years)		
18–24	107	32.4
25–34	143	43.3
35–44	57	17.3
≥ 45	23	7.0
Sex		
Male	193	58.5
Female	137	41.5
Marital status		
Single	59	17.9
Married	257	77.9
Divorced	14	4.2
Religion		
Muslim	317	96.1
Orthodox/protestant	13	3.9
Ethnicity		
Oromo	318	96.4
Amhara/Guraghe)	12	3.6
Main occupation		
Farmer	171	51.8
Housewife	79	23.9
Student	34	10.4
Merchant	28	8.5
Other (employee/laborer)	18	2.4
Educational level		
No formal education	214	64.8
Primary education	83	25.2
Secondary and above	33	10.0
Family size		
>5	257	77.9
≤5	73	22.1
Average monthly income in Ethiopian birr		
<2000	151	45.8
2000–3000	89	27.0
>3000	90	27.3

### Nutrition and diet information

Nearly three-fourth (73.6%) of the participants received nutrition care and support during TB treatment. The majority (81.8%) of adult TB patients had nutritional counseling. Mean individual dietary diversity score of the participants was 7.60 ± 2.56. Nearly two-third (63.0%) of the participants had consumed less than seven food groups in the last 24 h prior to interview. Cereals (98.5%) were the most consumed food-variety while fish was the least consumed (1.5%). Around two-third (69.1%) of the participants was living in food insecure household or family (Table 2).

**Table 2:** Dietary characteristics of adult tuberculosis patients on follow up at public health facilities in Haramaya district, eastern Ethiopia, 2021(n = 330)

Characteristic	N	%
Main food source of the family		
Own	53	16.1
Purchase/market	77	23.3
Both	200	60.6
Nutrition care and support during TB treatment		
Yes	243	73.6
No	87	26.4
Dietary counseling during TB treatment		
Yes	270	81.8
No	60	18.2
Feeding pattern (unit/place)		
Home	202	61.2
Outside	128	38.8
Individual dietary diversity score (IDDS)		
<7 (low)	208	63.0
≥7 (high)	122	37.0
The 14 food groups used to compute IDDS(the 24 h recall method)		
Cereals	325	98.5
Vitamin A rich Vegetables	264	80.0
White roots and tubers	223	67.6
Dark green leafy vegetables	211	63.9
Other Vegetables	226	68.5
Vitamin A rich Fruits	150	45.5
Other fruits	125	37.9
Organ meats	123	37.5
Flesh meats	94	28.5
Eggs	165	50.0
Fish	5	1.5
Legumes, nuts and seeds	148	44.8
Milk and milk products	236	71.5
Oil and fats	214	68.5
Household food security status		
Insecure	228	69.1
Secure	102	30.9

*N* number, *TB *tuberculosis, *IDDS *individual dietary diversity score

### Clinical and lifestyle factors

More than three-fourth (79.1%) of adult TB patients used at least one substance (Khat or Cigarette or Alcohol). Forty-three (13.0%) and 62(18.8%) adult TB patients had feeding problem and family history of TB, respectively. The majority (87.6%) of participants were new adult TB patients. Only seven adult TB patients had HIV co-infection and few (8.9%) participants had co-morbidity at time of registration (Table 3).

**Table 3:** Clinical and lifestyle factors of adult tuberculosis patients on follow up at public health facilities of Haramaya district, eastern Ethiopia, 2021 (n = 330)

Characteristic	N	%
Physical activity of ≥ 30 min exercise per week		
0 day per week	100	30.3
1–3 days per week	132	40.0
≥ 4 days per week	98	29.7
Substances use		
Yes	261	79.1
No	69	20.9
If yes, specify the substances used (multiple response is possible)		
Cigarette smoking		
Yes	16	4.8
No	314	95.2
Alcohol drinking	1	0.3
Yes		
No	329	99.7
Khat chewing		
Yes	261	79.1
No	69	20.9
Eating problem	43	13.0
Yes		
No	287	87.0
Family history of TB	62	18.8
Yes		
No	268	81.2
Type of TB treatment	289	87.6
New		
Retreatment	41	12.4
HIV co-infection	7	2.1
Yes		
No	323	97.9
Duration (phase) of anti-TB treatment (in months)	54	16.4
≤ 2 /Intensive		
>2/Continuation	276	83.6
Presence of co-morbidity	28	8.5
Yes		
No	302	91.5
If yes, which type? (multiple response is possible)	10	35.7
Diabetes mellitus		
Yes		
No	14	64.3
Asthma	4	14.3
Yes		
No	24	85.7
Heart diseases	1	3.5
Yes		
No	27	96.5

HIV=Human immunodeficiency virus, N=Number, TB=Tuberculosis

### Environmental factors

More than two-third (68.2%) of the participants had latrine facility of which 65.8% pit latrine. Two hundred sixty-three (79.7%) participants had been collecting drinking water from protected source mainly protected well. All participants had living house and 238(72.1%) of the participants were living with domestic animal in a house (Table 4).

**Table 4:** Environmental related factors among adult tuberculosis patients in public health facilities at Haramaya district, eastern Ethiopia, 2021 (n = 330)

Characteristic	N	%
Latrine possession		
Yes	225	68.2
No	105	31.8
If yes, which type of latrine? (n = 225)		
Pit latrine	148	65.8
VIP latrine	77	32.8
Drinking water source status		
Protected	263	79.7
Unprotected	67	20.3
Time taken to fetch drinking water from a source		
<5 min	41	12.4
5–10 min	115	34.8
11–30 min	159	48.2
>30 min	15	4.5
Living house possession		
Yes	330	100.0
No	0	-
Domestic animal in living house		
Yes	238	72.1
No	92	27.9

VIP, Ventilated improved pit

## Magnitude of undernutrition

The magnitude of undernutrition was 43.6% (95% CI 38.2%, 49.1%). Proportions of severe, moderate and mild undernutrition was 11.8%, 12.4%, 19.4%, respectively.

## Factors associated with undernutrition

In the bivariable analysis, individual dietary diversity score, family size, latrine possession, type of TB, duration of anti-TB treatment and family history of TB were significantly associated with undernutrition of adult TB patients at P < 0.05 and while other variables such as age, education status, monthly income, eating problem, nutritional counseling and co-morbidity were not significant at P > 0.05, but considered as a candidate variable in multivariable model at P-value < 0.25 (Table 5).

**Table 5:** Bivariable and multivariable logistic regression of factors associated with Under nutrition among adult TB patients attending public health facilities in Haramaya district, eastern Ethiopia, 2021 (n = 330)

Associated factors	Undernutrition		COR (95% CI)	AOR (95% CI)
	Yes, n (%)	No, n (%)		
Age (in years)				
< 25	57(53.3)	50(46.7)	2.14(0.84, 5.46)	**4.12(1.36, 12.51)**
25–34	59(41.3)	84(58.7)	1.32(0.53, 3.31)	1.98(0.69, 5.72)
35–44	20(35.1)	37(64.9)	1.10(0.37, 2.80)	1.88(0.60, 5.88)
> 44	8(34.8)	14(65.2)	1	1
Education status				
No formal education	101(47.2)	113(52.9)	1.52(0.96, 2.41)	**1.76(1.01, 3.08)**
Formal education	43(37.1)	73(62.9)	1	1
Family size				
> 5	41(56.2)	32(43.8)	1.92(1.13, 3.24)	**2.62(1.43, 4.82)**
≤ 5	103(40.1)	154(59.9)	1	1
Monthly income (in ETB)				
< 2000	74(49.0)	77(51.0)	1.66(0.97, 2.83)	1.45(0.77, 2.73)
2000–3000	37(41.6)	52(58.4)	1.23(0.67, 2.24)	1.21(0.59, 2.47)
> 3000	33(36.7)	57(63.3)	1	1
Nutritional counseling				
No	32(53.3)	28(46.7)	1.61(0.92, 2.83)	1.85(0.99, 3.45)
Yes	112(41.5)	158(58.5)	1	1
Dietary diversity				
Low (< 7)	71(58.2)	51(41.8)	2.58(1.63, 4.07)	**2.96(1.75, 4.99)**
High (≥ 7)	73(35.1)	135(64.9)	1	1
Eating problem				
Yes	23(53.5)	20(46.5)	1.38(0.86, 2.22)	1.21(0.56, 2.57)
No	121(42.2)	166(57.8)	1	1
Latrine possession				
No	59(56.2)	46(43.8)	2.11(1.32, 3.38)	**2.14(1.26, 3.65)**
Yes	85(37.8)	140(62.2)	1	1
Family history of TB				
Yes	34(54.8)	28(45.2)	1.74(1.01, 3.04)	1.72(0.90, 3.28)
No	110(41.0)	158(59.0)	1	1
Type of anti-TB treatment				
Retreatment	25(61.0)	16(39.0)	2.23(1.14, 4.36)	**2.56(1.19, 5.54)**
New	119(41.2)	170(58.8)	1	1
Phase of anti-TB treatment				
Intensive phase	34(63.0)	20(37.0)	2.57(1.40, 4.69)	**3.18(1.62, 6.25)**
Continuation phase	110(39.9)	166(60.1)	1	1
Co-morbidity				
Yes	17(60.7)	11(29.3)	2.13(0.96, 4.70)	1.58(0.64, 3.90)
No	127(42.1)	175(57.9)	1	1

Independent pridictors of the out come variables are shown in bold

AOR = Adjusted Odds Ratio, COR = Crude Odds Ratio, TB = Tuberculosis, ETB: Ethiopian Birr

In multivariable logistic regression analysis age, educational status, family size, dietary diversity, latrine possession, type of anti-TB treatment and duration of taking anti-TB treatment found significantly associated with undernutrition. The odds of undernutrition was four times [AOR = 4.12, 95% CI (1.36, 12.51)] higher among adult TB patients who aged less than 25 years compared to those aged ≥ 45 years. The odds of undernutrition was nearly two [AOR = 1.76, 95% CI (1.01, 3.08)] times higher among adult TB patients who had no formal education compared to those who had it. The odds of undernutrition was 2.62 times [AOR = 2.62, 95% CI: (1.43, 4.82)] higher among adult TB patients living in large family size (> 5) compared to those who had small (≤ 5). The odds of undernutrition was nearly three [AOR = 2.96, 95% CI (1.75, 4.99)] times higher among adult TB patients who had low individual dietary diversity compared to those who had high individual dietary diversity. The odds of undernutrition was two times [AOR = 2.14, 95% CI (1.26, 3.65)] higher among adult TB patients had latrine compared to those who hadn’t. Odds of undernutrition was 2.56 times [AOR = 2.56, 95% CI (1.19, 5.54)] and 3.18 times [AOR = 3.18, 95% CI (1.62, 6.25)] higher among adult TB patients who were previously treated and being on intensive phase of anti-TB treatment compared to their counter parts respectively (Table 5).

## Discussion

In this study, the magnitude of undernutrition and related determinants were investigated in adult TB patients receiving follow-up treatment at public health facilities in the Haramaya district of eastern Ethiopia. As a result, the proportion of adult TB patients who were undernourished was 43.6%. Age between 18 and 24 years, no formal education, large families (more than five people), low dietary diversity, a lack of latrines, having been previously treated for TB, and being in the intense phase of treatment were all significantly associated to undernutrition in adult TB patients.

Four out of ten (43.6%) adult TB patients in this study had undernutrition, indicating that the study area had a high prevalence of undernutrition. This result is consistent with studies from Hosanna, southern Ethiopia (38.9%), Addis Ababa, Ethiopia (39.7%) and Kenya (43%) [14, 20, 21]. However, it is lower than studies conducted in Tigray, northern Ethiopia (51.2%) [22], Adama, central Ethiopia (53%) [23], Bale, southeast Ethiopia (63.2%) [24], Metema district, northwest Ethiopia (51%) [25] and Amhara, northern Ethiopia (55.6%) [26]. Additionally, it is considerably lower than studies conducted in Tripura, India (66%) [11] and Thyolo, southern Malawi (57%) [27]. This discrepancy may be brought about by variances in participant sociodemographic and economic characteristics, research dates and locations, participant lifestyle and dietary habits, and the different sample sizes that were used [20, 21, 26]. Additionally, some studies measured TB patients’ BMIs at the time of diagnosis rather than when they were taking anti-TB medications while they were ill and in a state of critical undernourishment, which could exaggerate the expected level of undernutrition [10]. While in other studies, the outcome variable, BMI, was measured at the end of anti-TB treatment (treatment completion or cure), when participants had already recovered from all illnesses and malnutrition and were at the state of critical undernourishment. This may have underestimated the level of undernutrition [22]. Beside the observed difference could also be due to the difference in inclusion criteria used for the study: For, example, few studies included only new adult TB patients and other studies included PTB patients [28]. TB patients who are pregnant were also included in other studies that, increase or underestimate the degree of undernutrition among TB patients.

In this study, the odds of undernutrition were four folds higher among adult TB patients aged between 18 and 24 years compare to those aged 45 years and above. The observed finding may have resulted from the fact that young people (15–24 years old) typically lack the necessary experience to promptly and appropriately seek out available healthcare services [29, 30]. Moreover, they had poor eating habits, such as eating outside home and skipping meals, despite the fact that their bodies needed more and more nutrients for growth and development [31]. Youths are also more likely to have substance use disorders (Khat, alcohol, smoking), infectious diseases like HIV, and a general lack of knowledge about nutrition and dietary feeding [32–34]. All of these factors could increase the risk of malnutrition among adult TB patients, just as they did in healthy youths.

In this study, the prevalence of undernutrition was nearly twice as high among adult TB patients without a formal education as it was in those who had it. This finding is similar with the study done in Amhara, northern Ethiopia [26] and Gulbarga, India [34] that showed illiterate patients were two-folds higher risk of malnourished as compared to literates.

In this study, there was a 2.62 times higher risk of undernutrition among adult TB patients who lived in large families (> 5). This result is consistent with research carried out in Hosanna, southern Ethiopia (three times higher ) [14], and northern Ethiopia (15.75 folds higher) [35]. The difference seen in the later study could be due to the difference in the study setting.

When compared to adult TB patients who had high individual dietary diversity, the odds of undernutrition were about three times greater for those with low individual dietary diversity. It is supported by the fact that tuberculosis patients lack essential nutrients such as vitamins, resulting in malnutrition and a weakened immune system, increasing the risk of infection.  Low  dietary diversity also leads to micronutrient deficiency due to increased metabolic demands and decreased intake of micronutrients, which worsens diseases and delays recovery by suppressing immunity. Furthermore, there is a strong link between micronutrients and macronutrients and the prevention, treatment, and control of tuberculosis disease, but proper nutrition is essential for immune system development [36].

The odds of undernutrition was two times higher among adult TB patients who hadn’t latrine in their compound compared to those who had. This finding, similar to that found in the study conducted in Amhara region, northern Ethiopia [26], may be attributable to the low socioeconomical status. Indeed, poor hygienic conditions could facilitate the occurrence of infectious diseases through fecal contamination of soil, water, food, or other sources. Such communicable diseases lead to reduction in food intake, obstruction of nutritional absorption, and depletion of nutrients required to support the host development, contributing to undernutrition [35]. Thus, it is unlikely to solve the problem of undernutrition in TB patients only through the administration of appropriate medication if healthy environmental conditions are not ensured [37].

Malnutrition was 2.56 times higher among adult TB patients who had history of TB. This is supported by study conducted in secondary hospitals in Chhattisgarh state, central India [38]. Additionally, it is supported by the fact that a person with TB has a higher energy requirement, which frequently results in a TB patient losing a lot of weight, which might exacerbate acute undernutrition. In fact, over half of TB patients are malnourished when they are diagnosed with tuberculosis. This includes those around 17% of patients who are with severe malnutrition. Moreover, TB infection usually develops in those with a weakened immunity it usually promote poor health and contracting other secondary infection that increase the risk of malnutrition and history of TB, like other infectious diseases, is likely to increase energy requirement [39].

Malnutrition was three folds higher among adult TB patients who were on intensive phase of anti-TB medication compared to those who were on continuation phase. This finding is similar with the study done in Addis Ababa [20]. This may be explained by the fact that patients who took anti-tuberculosis drugs for up to two months were anticipating starting to recover from the illness when there were less episodes of vomiting, nausea, and loss of appetite.

As regards weaknesses, the design used in this study doesn’t allow to show causal relationships between predictors and outcome variables. Moreover, dietary diversity score and household food security were assessed employing a 24-hour and 30-day recall, respectively. Since both tools accuracy largely depends on the respondent’s memory, they may be subject to recall biases.

## Conclusion

In this study, the prevalence of undernutrition among adult TB patients was high. Age, educational status, family size, dietary diversity, latrine possession, being on the retreatment and intensive phase of anti-TB drug medications were factors significantly associated with undernutrition among adult TB patients. Based on the findings of this study the following recommendations are forwarded. Regional, Zonal and Wareda health office should focus on educating youths on preventive intervention measures to prevent malnutrition as integral component of TB treatment. Facilities those providing directly observed treatment of TB patient (health center, hospitals) are implementing regular undernutrition screening program for TB patients. Health extension worker should work hard on nutrition education at community level for increasing awareness on risk factors of undernutrition.

## Data Availability

The data sets generated during and/or analyzed during the current study are available from the corresponding authors on reasonable request.
